# Role of Nanomaterials in the Fabrication of bioNEMS/MEMS for Biomedical Applications and towards Pioneering Food Waste Utilisation

**DOI:** 10.3390/nano12224025

**Published:** 2022-11-16

**Authors:** Nuraina Anisa Dahlan, Aung Thiha, Fatimah Ibrahim, Lazar Milić, Shalini Muniandy, Nurul Fauzani Jamaluddin, Bojan Petrović, Sanja Kojić, Goran M. Stojanović

**Affiliations:** 1 Department of Biomedical Engineering, Faculty of Engineering, Universiti Malaya, Kuala Lumpur 50603, Malaysia; 2 Centre for Innovation in Medical Engineering (CIME), Department of Biomedical Engineering, Faculty of Engineering, Universiti Malaya, Kuala Lumpur 50603, Malaysia; 3 Centre for Printable Electronics, Universiti Malaya, Kuala Lumpur 50603, Malaysia; 4 Faculty of Technical Sciences, University of Novi Sad, T. Dositeja Obradovića 6, 21000 Novi Sad, Serbia; 5 Faculty of Medicine, University of Novi Sad, Hajduk Veljkova 3, 21000 Novi Sad, Serbia

**Keywords:** nanomaterials, biomedical nanoelectromechanical systems (bioNEMS), biomedical microelectromechanical systems (bioMEMS), drug delivery system, point-of-care

## Abstract

bioNEMS/MEMS has emerged as an innovative technology for the miniaturisation of biomedical devices with high precision and rapid processing since its first R&D breakthrough in the 1980s. To date, several organic including food waste derived nanomaterials and inorganic nanomaterials (e.g., carbon nanotubes, graphene, silica, gold, and magnetic nanoparticles) have steered the development of high-throughput and sensitive bioNEMS/MEMS-based biosensors, actuator systems, drug delivery systems and implantable/wearable sensors with desirable biomedical properties. Turning food waste into valuable nanomaterials is potential groundbreaking research in this growing field of bioMEMS/NEMS. This review aspires to communicate recent progress in organic and inorganic nanomaterials based bioNEMS/MEMS for biomedical applications, comprehensively discussing nanomaterials criteria and their prospects as ideal tools for biomedical devices. We discuss clinical applications for diagnostic, monitoring, and therapeutic applications as well as the technological potential for cell manipulation (i.e., sorting, separation, and patterning technology). In addition, current in vitro and in vivo assessments of promising nanomaterials-based biomedical devices will be discussed in this review. Finally, this review also looked at the most recent state-of-the-art knowledge on Internet of Things (IoT) applications such as nanosensors, nanoantennas, nanoprocessors, and nanobattery.

## 1. Introduction

Biological (or biomedical) applications of nano-/micro-electro-mechanical systems (bioNEMS/MEMS) are emerging advanced devices for rapid, sensitive, and real-time analysis of biological samples. Micro and nanofabrication started with bulk and surface micromachining for silicon in the electronic industry. The rapid development of single-crystal silicon using MEMS technology has led to an interest in employing glass and polymer-based materials to fabricate MEMS devices. As a result, this technology branched out to cater to other industries such as biomedical industries, which later formed the foundation for bioNEMS/MEMS technology [1,2]. bioNEMS/MEMS devices are expected to perform sample screening and analysis cycles in the millisecond to picosecond range, necessitating careful consideration of factors such as device design, sensor sensitivity, and material selection. Nanomaterials-based bioNEMS/MEMS have found potential in a myriad of biomedical applications, from drug delivery systems, cell manipulation (e.g., sorting–separation technology and cell patterning) to point-of-care testing (e.g., surgical sensors, implant sensors for tissue engineering applications, and rapid home monitoring devices) [3,4,5,6]. Hence, the development of high-throughput nanomaterials-based bioNEMS/MEMS devices is needed.

**Figure 1:**
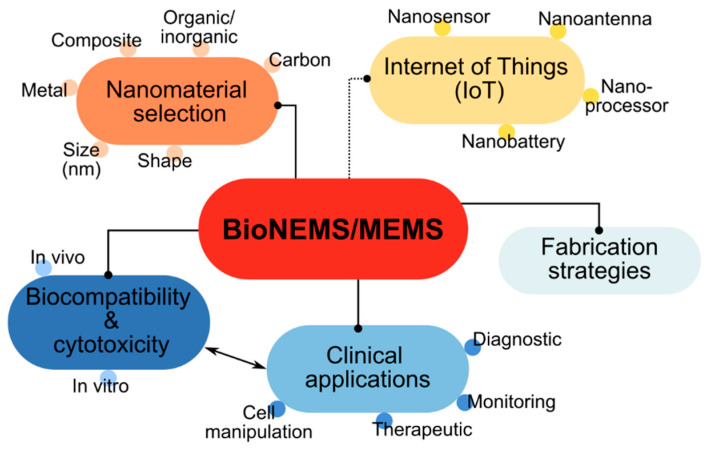
Development trends and clinical applications of nanomaterials-based bioNEMS/MEMS devices and future opportunities for integration with Internet of Things (IoT) technology.

The primary goal of this review is to explore the progress of nanotechnology and bioNEMS/MEMS by focusing on the fabrication strategies and clinical applications that have rapidly evolved in the past years, as summarised in Figure 1. In this framework, we discuss the developmental trend of nanomaterials-based bioNEMS/MEMS and the prospects for future biomedical devices. The amalgamation of multidisciplinary fields between engineering, materials science, chemistry, biology, and computer science has contributed to the rapid advancement of this technology. In this instance, the integration of bioNEMS/MEMS to the Internet of Things (IoT) technology could be realised in the near future. Therefore, we also discuss the current state-of-art of IoT platforms and their potential to integrate information into bioNEMS/MEMS nanodevices. Lastly, challenges and future opportunities for further development of nanomaterials-based bioNEMS/MEMS will be thoroughly highlighted in this review.

## 2. Overview of Nanomaterials for bioNEMS/MEMS Applications

### 2.1. Classification of Nanomaterials

Nanomaterials in biomedical applications enable the miniaturisation of bioNEMS/MEMS with multifunctional capabilities, which facilitates control and regulation of the biological environment [7,8]. The miniaturisation of devices is important to allow their operation in a specific area for a selected duration of time. Materials at the nanoscale range exhibit outstanding electrical, mechanical, chemical, and optical properties and an increase in the surface area of a material also enhances its catalytic ability [9,10]. However, the biocompatibility of nanomaterials-based bioNEMS/MEMS such as biosensors, actuation systems, drug delivery systems and smart stents is an important concern to prevent the toxicity and loss of functionality of the device in both in vitro and in vivo conditions [11]. Some of the important criteria of nanomaterials that are promising for the fabrication of bioNEMS/MEMS are their fascinating tuneable electronic, mechanical, and optical properties [12,13]. Furthermore, nanomaterials also promote the adhesion between the substrate and biological molecular layer and the substrate, which enables the tunnelling-based charge transport of the nanocomposites [14]. In addition to that, other properties such as chemical stability, thermal stability, heat conductance, porosity, and surface area are also important features of the nanomaterials-based bioNEMS/MEMS [15,16].

**Figure 2:**
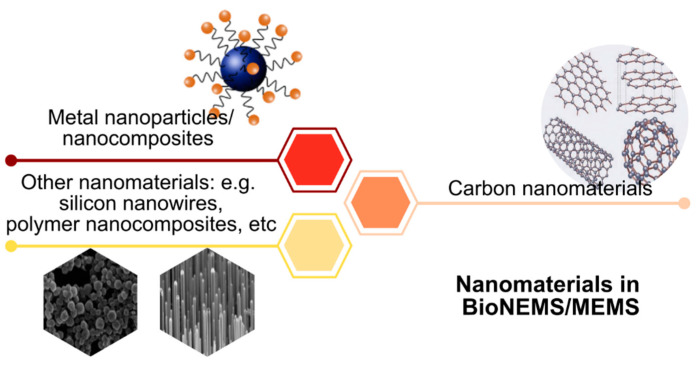
Types of inorganic nanomaterials developed using bioNEMS/MEMS techniques for biomedical applications.

Metal nanoparticles, carbon nanomaterials including polymer nanocomposites, are the most commonly adopted nanomaterials for the fabrication of bioNEMS/MEMS (Figure 2) [17,18]. The heterogeneous integration of bioNEMS/MEMS devices with nanomaterials has created a breakthrough in the biomedical field for applications such as sensing, diagnostics, imaging, and therapeutics. The nanomaterial-bioNEMS/MEMS developed for drug delivery applications include micropumps, microneedles, and micro-reservoirs to achieve targeted drug delivery. The nanomaterials-based micro/nanoneedles are small-scale bioNEMS/MEMS devices that possess unique properties whereby they can be modified for selective transport of drug molecules, and the superior electronic properties of nanomaterials enable the control of the delivery by applying an electric field [11]. For example, a novel approach of integrating carbon nanotube (CNT) nanofilters with SU-8 microneedle array devices was fabricated for transdermal drug delivery through the lithography process [19]. The fabricated device is capable of penetrating the stratum corneum layer and with the help of CNT, the drug molecules are selectively delivered to specific tissues with an applied electric field. Similarly, for drug delivery into the tissue, a nanosized drug delivery vehicle bioNEMS/MEMS that employs the micro-nano reservoirs concept was used [20]. Paclitaxel drug delivery into human glioblastoma cells and human umbilical vein endothelial cells tissues was achieved with the help of a nanomaterial coated biocompatible co-polymer block of poly(lactide)-poly(ethylene glycol) (PLA-PEG). The co-polymer was found to reduce the in-vitro toxicity and the surface properties of nanomaterials prevent tissue aggregation and improve the drug loading to increase therapeutic efficacy.

In the field of biosensing, the nanomaterial-coated bioNEMS/MEMS plays a significant role in the biomedical field due to some unique features such as portability, fast response, and high sensitivity. The unique biological and physical properties of nanomaterials enable target biomolecule identification and cause–effect transduction of an electronic signal in bioNEMS/MEMS biosensors [11]. The integration of nanomaterials in the system allows the miniaturisation of the system into a single chip to facilitate the fabrication of point-of-care devices. A study by micro-nanocantilevers increased immobilisation of the cholesterol oxidase enzyme for sensitive detection of cholesterol levels up to 100 femto molar within a short period of response time. Similarly, Sharma, et al. [21] explored the effect of gold nanoparticles on the electrical conductivity, electroactive surface area and electrochemical activity of nano-sized carbon interdigitated electrodes. This amperometric MEMS biosensor exhibited a wide sensing range (0.005–10 mM), high selectivity and high sensitivity with a limit of detection (LOD) of ~1.28 µM for cholesterol detection.

### 2.2. Nanomaterials Synthesised from Food Waste

**Figure 3:**
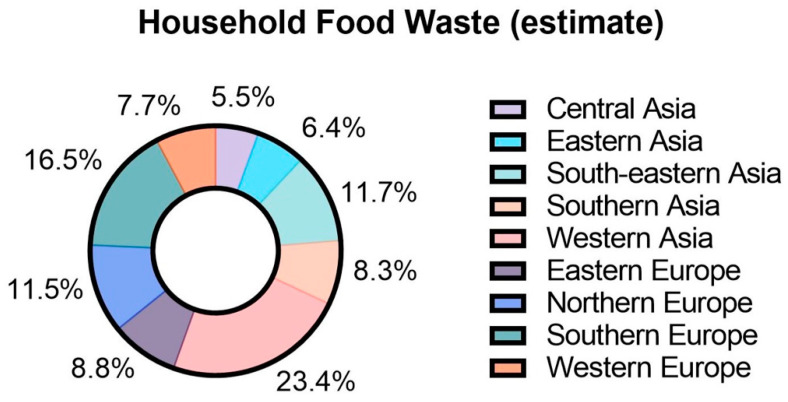
Household food waste in Asia and Europe countries published in Food Waste Index Report 2021 [27].

In past years, a plethora of nanoparticles have been produced through various techniques and adopted to advance technologies for biomedical applications [22]. With the advancement of knowledge in the field of materials science and engineering, researchers are keener on ensuring the sustainability of the nanomaterials production processes. Through these initiatives, many alternative inputs for nanomaterials production as well as green synthesis techniques were introduced widely. Rapid urbanisation led to a massive increase in food waste or food-derived waste which includes agricultural waste [23]. Around 1.3 billion tons per year of edible food produced for human consumption were wasted globally. The wastage of food starts from the initial stage of agricultural production down to the final household consumption [24]. For medium- and high-income countries, food wastage is high at the final consumer stage, whereas in low-income countries the wastage occurs initial or middle stage of the food supply [25]. Moreover, in Europe and North America, food wastage accounts for 280–300 kg/year, which is equivalent to 95–115 kg/year per capita. However, in Sub-Saharan Africa and South/Southeast Asia, food wastage accounts for 120–170 kg/year, which is equivalent to only 6–11 kg/year per capita [26]. According to the recent food waste index published in 2021 as summarised in Figure 3, European countries produce approximately 3400 kg/capita/year, whereas Asia is one of the highest contributors of food waste, producing more than 4300 kg/capita/year of household food waste [27].

Aside from the startling statistics, the negative repercussions of wasted food on society take many other forms, ranging from immediate financial losses to environmental pollution including air and land contamination. Products with low and negative values (waste) can be converted into useful resources. Its utilization as a resource for the production of high-value materials is a profitable route to improving sustainability and lowering financial expenditure of many industries and scientific disciplines including biomedicine and nanomaterials [28]. The wastes can be utilized to produce valuable nanomaterials such as carbon-based nanomaterials, silver, zinc, gold and chitosan that can be used for biomedical applications as they add integrated functionalities to the bioNEMS/MEMS devices [23,29,30]. Interestingly, food wastes are a valuable source for the green synthesis of nanomaterials due to their low toxicity, cost-effectiveness, nano-size, and high stability properties. These features of nanomaterials are important for integration with bioNEMS/MEMS especially, for therapeutic and diagnostic purposes as the system must be biocompatible as their use should not cause cell toxicity [31]. Due to the organic nature of most of the food waste, the nanomaterials can be synthesized using green ways to complement the traditional synthesis method such as chemical, hydrothermal, and high-pressure treatments, which can be toxic due to the use of adverse chemicals and that limits its biomedical applications. Nanomaterials from food waste are often synthesized using the pyrolysis method, carbonization, thermal decomposition and other chemical-free green synthesis methods [32,33,34,35].

Up to now, very limited studies reported on utilizing food waste such as eggshells, peanut shells, coconut shells and coconut noir to fabricate MEMS devices [36,37,38]. Despite that, with the aid of two different kinds of agricultural waste, especially coconut coir and coconut shell, the method for converting waste into high-value capacitor-reduced graphene oxide electrodes has been proposed [37]. It has also been shown that negative value peanut shells, a common agricultural waste, can be converted into graphene-like nanosheets consisting of several layers [38]. Because of the low cost and enormous amount, which can help in addressing global problems such as water and energy crises, this food waste reuse strategy has enormous significance for the upcoming management of food waste and material science. It also holds truly excellent for a sustainable society. In this view, food waste-based bioNEMS/MEMS could open a new research niche following explosive interests in food waste-based nanomaterials. Repurposing food waste into innovative products is one of the promising circular economy approaches in an attempt to tackle the food waste problem. Therefore, the exploration of new research niches could be a blueprint to achieve sustainable food waste management in future. Furthermore, this new research area is in alignment with the 2030 Sustainable Development Goals set by the United Nations General Assembly in its effort to reduce 50% of food waste by 2030 [31,39].

## 3. Perspective on Nanotechnology as an Enabling Tool for the Design of New Functional Materials and Devices for bioNEMS/MEMS in Medical Applications

The integration of functional nanomaterials in the fabrication of bioNEMS/MEMS created a breakthrough in the biomedical field. Nanomaterials introduce new features and increase the overall performance in terms of sensitivity, portability, specificity, and throughput without affecting the economic feasibility of the system. Nanomaterials with bioNEMS/MEMS improve the overall mechanical properties of the system and allow the researchers to better understand the cellular interactions at the molecular level [40,41]. Moreover, nanomaterials (e.g., carbon-based nanoparticles, graphene nanosheets, silver, zinc and gold nanoparticles) in biomedical applications provide good control and regulation of the biological environment because the micro-sized devices can be tuned to acquire controlled multifunctional capabilities.

Metal nanoparticles are used in the fabrication of bioNEMS/MEMS for numerous applications due to their ease of scalability, high surface-to-volume ratio and good optical properties [42,43]. In particular, gold nanoparticles are widely used in biomedical applications mainly due to their low toxicity, good biocompatibility, tuneable surface plasmon resonance (SPR), easy biofunctionalization and detection [44]. MEMS are usually coated with gold nanoparticles which are photoresponsive nanomaterials that create laser-based actuators for applications such as studying the effect of mechanical forces on cells, and remotely triggering cardiac muscle stimulators [45]. The metal nanoparticles-MEMS hybrid acquires good biocompatibility which helps create infrared light-driven cellular scale mechanical actuators that can be distributed throughout various types of tissue samples [46]. For instance, Schlicke, et al. [47] fabricated micro- and nanoelectromechanical systems with freestanding organically cross-linked gold nanoparticle (AuNPs) membranes with a thickness of 29–45 nm deposited through lithographic patterning in a SU-8 resist. This finding can be used for the fabrication of electrostatic actuators due to the conductive and flexible nature of the hybrid system and can be tailored for different applications by tuning the particle size or structure of the cross-linker for mechanical, electrical, and optical properties based on the different requirements.

Currently, bioNEMS/MEMS devices rely on the usage of silicon, but it has poor mechanical and thermal stability, which creates a demand for more promising materials to be integrated into the fabrication of the system for biomedical applications [48]. Carbon nanomaterials such as graphene, nanodiamonds, and carbon nanotubes are some of the promising alternatives due to some remarkable properties such as high Young’s modulus, hydrophobic surface, low mass, tailorable electronic configuration and high thermal conductivity [49]. Traditional bioNEMS/MEMS face major hurdles such as contamination diffusion barrier properties, high variability, low yields, low physical and chemical stability, tribology and wear rates and low reproducibility of surface functionalization which limits their commercial applications [50]. Thus, with integration with carbon nanomaterials, commercial feasibility and scalability are made possible.

Graphene is a two-dimensional (2D) material with high strength and stiffness that can be used in bioNEMS and MEMS applications [51]. Graphene has high mechanical strength, high carrier mobility, low density, and remarkable mechanical and electrical properties which makes it a suitable transducer candidate for beams and membranes in bioNEMS/MEMS applications [52]. Moreover, it also provides substantial device scalability without compromising the performance of the device. Its excellent electrical conductivity enables integrated electrical transduction and its planar geometry allows itself easy compatibility with patterning using a standard lithographic process [53]. For biomedical applications, graphene is mostly used for sensing purposes. For instance, Rahman, et al. [54] researched the feasibility of applying graphene membrane as a pressure sensor diaphragm in the MEMS piezoresistive pressure sensor for sensitive intracranial pressure monitoring. The study reported that the proposed graphene-based MEMS device structure is suitable to detect pressure-induced strain even at low-pressure applications. Moreover, Khan and Song [55] fabricated graphene field-effect transistor (GFETs)-based bioMEMS devices for the detection of interleukin-6 (IL-6) protein biomarkers. In this study, pyrene-tagged deoxyribonucleic acid (DNA) aptamers were immobilised on the sensing platform easily due to the presence of graphene which aids the π–π stacking bonding with the negatively charged DNA aptamer in the presence of negative electric field. Graphene increased the immobilization rate and the surface coverage for a more sensitive IL-6 protein detection up to the picomolar range. Graphene has explored significant potential, especially as the negative piezoconductive effect of mono-layer graphene which can be used for flexible sensors for electronic skin and wearable devices [56]. Chen, et al. [57] developed a nanowires/graphene heterostructure-based pressure sensor for static pressure measurement. The synergistic effect between the strain-induced polarisation charges of piezoelectric nanowire and the high carrier mobility of graphene achieved sensitivity for static pressure measurement up to 1.06 × 10^2^ kPa of within 5–7 ms response time. The nanowires/graphene heterostructure-based device design developed in this study is suitable for wearable human health inspection.

Carbon nanotubes offer great potential for electronic and biomedical applications owing to their outstanding mechanical, electrical, optoelectrical, thermal, and physical solid-state surface properties [58,59]. With a controlled synthesis condition, structural properties (chirality, semi-conducting/metallic properties, single or multi-walled), and size (diameter/length), CNT extend their applicability to bioNEMS/MEMS [50]. With these advances in the area of biomolecule–CNT hybrid systems, many potential applications within the biomedical field can be explored. The CNT can also be integrated into a carbon microstructure using various MEMS fabrication techniques. The large active surface area of CNT-modified carbon microstructures shows great potential and performance in biosensing. Xi, et al. [60] studied the efficiency and performance of a multi-walled CNT-modified C-MEMS electrode for the detection of glucose. In this study, the glucose oxidase was immobilised in polymerised polypyrrole film onto the CNT carbon microstructures in which the presence of CNT leads to increased electroactive sites for enzyme loading and facilitates rapid electron transfer rate. This CNT-based MEMS amperometric glucose biosensor results in the sensitive detection of glucose with a linear detection range of 5 to 80 mM and a limit of detection of 97.3 mA M^−1^ cm^−2^ within 10 s of response time. Moreover, MEMS technology also paves a way for the fabrication of microelectrode arrays (MEA) with uniform electrochemical characteristics for various biomedical applications. Xu, et al. [61] developed an MEA-based bioMEMS device for electrochemical detection of 5-hydroxytryptamine and dopamine for neural function analysis at both the single cell and network levels. The microelectrodes were coated with chitosan multi-walled carbon nanotubes (MWCNT) hybrid through electrochemical deposition. This nanomaterial film allows high loading of electrochemically active (bio-) molecules and improves the electrochemical currents of diffusing electroactive species, which leads to high signals. The high catalytic activity of the hybrid film bioMEMS resulted in oxidation peak separation of the electroactive neurotransmitters along with improved current response with a detection range of 5 × 10^−6^ M to 2 × 10^−4^ M and 1 × 10^−5^ M to 3 × 10^−4^ M for dopamine and 5-hydroxytryptamine, respectively. Carbon nanotubes are also often coupled with polydimethylsiloxane as polymer nanocomposites for biomedical applications. For instance, these nanocomposites have been used for the fabrication of flexible pressure sensors to detect endovascular aneurysm repair problems [62]. The feasibility of CNT-based NEMS/MEMS has been proved by many researchers. This integration will result in the next generation of nanotransducers.

## 4. NEMS/MEMS Fabrication Technologies with Nanomaterials

**Figure 4:**
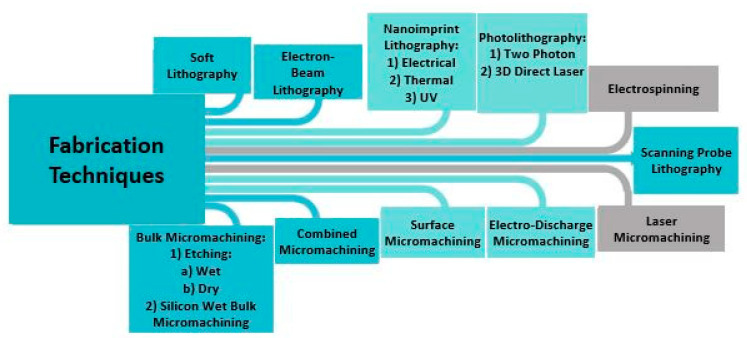
bioNEMS/MEMS fabrication strategies.

In this section, NEMS and MEMS fabrication technologies, which pertain to nanomaterial manipulation, will be described, as well as the ones which are most commonly used, such as bulk micromachining techniques. The aforementioned techniques are micromachining techniques, and the following ones will be described: photolithography, electron beam lithography, scanning probe lithography, soft lithography, nanoimprint lithography, bulk micromachining, surface micromachining, electro-discharged micromachining, laser micromachining and suspended carbon nanowire technique (Figure 4). In the process of fabrication these techniques can be used sequentially.

### 4.1. Photolithography

Photolithography, like other lithography processes, uses a substrate, most popularly silicon wafers, onto which a layer of coating is applied. Photoresists are coated on a layer of nitride, oxide, or any metal film that has been either deposited or grown on the aforementioned silicon wafer during the photolithography process. Depending on the type of photoresist, exposing it to light either removes or leaves the sensitive layer behind (positive or negative, respectively). As a result, the pattern is transferred from the overlaying mask to the photoresist [63]. Following that, other techniques such as etching are used. Photolithography can be used multiple times, layer by layer [64].

Photolithography, also known as optical lithography or ultraviolet (UV) lithography, is highly resistant to chemical agents, bases, and acids. It is also distinguished by its high photosensitivity to radiation. It has adequate adhesion to the surface of the substrate. Working resolution is limited by the diffraction of the used light and also the wavelength of the used radiation beam [65]. Photolithography’s rapid and selective UV-curing allows for faster fabrication than soft lithography. In light of this, there has been an increase in the preparation of preceramic photoresists [63].

As it is a relatively common method in practice, it has been beneficial in developing novel approaches to different types of sensors. Micro-machined Al shadow masks have been reported to be used in photolithography to create resonant sensors [66]. Furthermore, a three-step photolithographic process for releasing a resonator structure also has been proposed [66]. Sui, et al. [67], used photolithography to define the chromium (Cr) hard mask after the completion of chromium deposition with the goal of fabricating GaN/AIN. A different photolithography technique known as two-photon lithography has been used for manufacturing complex 3D structures on the micro- and nanoscale [68]. Three-dimensional direct laser lithography based on multiphoton absorption has also been used for nanoscale structure fabrication [69]. However, both photolithography and conventional lithography have been shown to be time-consuming processes; as a result, laser machining techniques are becoming more popular, overcoming this limitation [68].

Aside from directly fabricating NEMS/MEMS, photolithography is used as a fabrication step for cantilevers. UV lithography was used as the first step in the fabrication of cantilevers by Udara, et al. [70]. X-ray photolithography is used to create patterns in relatively thick photoresist layers [71].

### 4.2. Electron Beam Lithography

Aside from the variations discussed in the following paragraph, electron beam lithography (EBL) is a serial write patterning process that produces patterns as a direct result of a small spot of electrons being deflected across a substrate [72]. The previously mentioned process is mostly used in nanoscale pattern creation [73].

The advantages of EBL over other methods include the absence of a mask for pattern transfer [73]. Moreover, it gives precise control over the energy and dose of the electron beam, as well as accurate registration over small areas on a wafer and lower defect densities [64]. However, the application of EBL is constrained by its slow exposure speed, high cost, and resolution limitations caused by electron beam diffraction in solids [64,74].

### 4.3. Scanning Probe Lithography

Scanning probe lithography (SPL) is another type of atomic-scale write patterning process based on scanning tunnelling microscopy, which generates images by passing a conducting tip over a surface with a constant tunnelling current [64]. The advantage of SPL over EBL is that it has higher resolution and lower energy electrons with lateral scattering [64,75]. Aside from that, it has demonstrated other advantages over other lithographic processes. These include low costs, 3D patterning, high-quality patterning, and so on [76].

One significant disadvantage is that SPL, particularly mechanical SPL (m-SPL), which uses force to pattern, is relatively slow [76]. This can be overcome by using different types of SPL, such as thermal SPL [77]. Thermal SPL (t-SPL) uses concentrated heat to activate endothermic decomposition and evaporation of thermally sensitive resist [76].

SPL maximises friction-induced selective etching, which uses a diamond tip to scratch the surface of a resist. It has higher resolution and flexibility, making it a promising method for fabricating micro/nanostructures [78].

### 4.4. Soft Lithography

Soft lithography, which is primarily used for the fabrication of nano/microstructures, is a method that uses a master stamp to transfer the pattern onto a substrate using a special ink [64]. Because of its biocompatibility and low cost, polydimethylsiloxane (PDMS) has been most commonly used as a stamping material. Soft lithography is most commonly used for the development of microfluidic devices [79]. It is used for developing lab-on-chip and flexible electronics [80].

Soft lithography is typically comprised of six stages [81].

1. Soft mould creation;
2. Preparation of metal and ceramic slurries;
3. Filling of the soft mould with the prepared slurries;
4. Drying or curing and demoulding;
5. De-binding;
6. Sintering.

As previously stated, the soft lithography process is relatively slow due to the extensive steps and processes involved. As a result, photolithography is preferred for ease of fabrication [63]. There are five types of soft lithography, which include micro-moulding in capillaries, micro-transfer moulding, micro-contact printing, replica moulding and solvent-assisted micro-moulding [81].

### 4.5. Nanoimprint Lithography

Nanoimprint lithography (NIL) is a high resolution (more than 10 nm) and high efficiency lithography technique that employs a patterning technology that involves the deposition and exposure of a low viscosity resist onto a substrate [82]. This process utilises capillary action in which the fluid quickly flows into the lowered pattern mask and the resist will be exposed under UV light for curing. Finally, the pattern mask is removed, leaving the resist on the substrate [83]. NIL is used for nano structuring Si wafers [78], as well as crystalline silicon pillars [84].

Depending on the polymer characteristics, there are three families of NIL [85]:

1. Electrical NIL;
2. Thermal NIL;
3. UV NIL.

NIL has drawbacks despite being a high-efficiency, high-resolution method that is more widely used than other lithography techniques. The pattern stamp’s positioning is critical, and if there is a mechanical shift, the imprinting will be misaligned [75]. Aside from that, controlling defectivity and template life is difficult [74].

### 4.6. Bulk Micromachining

Bulk micromachining is a MEMS fabrication process that involves removing specific amounts of the silicon substrate to create the desired structure [71,80]. Etching is a significant subtype of bulk micromachining that will be discussed in detail below [71].

This method is most often performed by depositing a masking material layer and then patterning it by exposing the areas on the substrate which will be etched [86]. It has been used in a wide range of different MEMS applications (MEMS silicon accelerometers) [87], as well as cantilever fabrication [88,89]. Bulk micromachining techniques can be used along with other methods such as wafer bonding [90]. Aside from etching techniques, some bulk micromachining techniques are silicon wet bulk micromachining techniques.

1. *Silicon wet bulk micromachining*

Used mainly for the fabrication of cantilevers and cavities, silicon wet bulk micromachining is used in laboratories and the industry for MEMS applications [91]. It is used for surface texturing with the goal of minimising the reflectance of light [92].

2. *Etching*

Etching is the process of removing materials from a specified area [79]. It is divided into two groups: dry and wet etching. During the process of dry etching, microfeatures are achieved by milling through the usage of an ion beam or using reactive ion etching, whereas wet etching uses etchant solutions [81].

(a) Wet etching

The amount of etching of a substrate is controlled by parameters such as etching solution concentration, etching temperature, rate, and roughness [79], making wet etching highly modulable. The ability of batch fabrication is an important aspect of wet etching, resulting in it being widely used in the industry [91]. When it comes to NEMS/MEMS fabrication, wet etching can be used in creating anisotropic directional etches on crystalline materials [66] for the production of cantilevers [70], as well as in the fabrication of microfluidic devices [93].

(b) Dry etching

As the name implies, dry etching does not use any etchant solution. There are two types of dry etching, namely ion beam etching and reactive ion etching [79]. The ion beam etching produces smooth edges on curves, whereas reactive ion etching uses inductively coupled plasma (ICP-RIE) to remove the chromium oxide sacrificial layer at a cryogenic temperature [84,94]. Dry etching is a common method used in NEMS/MEMS applications. For example, this method was adapted in the process of making 3D silicon nanowires for FET biosensors [95]. With the goal of making PIN diodes, Schottky diodes, junction field-effect transistors (JFETs), and metal oxide semiconductor field-effect transistors (MOSFETs), the previously mentioned ICP-RIE is used [96]. ICP-RIE is also used in the fabrication of resonators for high-frequency bands [97]. Moreover, dry etching is used as a step-in fabrication of silicon-on-glass MEMS [98].

### 4.7. Surface Micromachining

As the opposite of bulk micromachining, surface micromachining techniques implore additive processes that utilise thin films. A sacrificial layer is usually used for creating a beam or membrane for moving parts. The most commonly used structural materials are polysilicon and metal [64]. It has various uses, mainly for NEMS/MEMS applications, capacitive MEMS accelerometer [87], and bulk acoustic resonators [66].

The drawback of using surface micromachining in comparison to bulk micromachining is the fact that there is a need for a post-process with the goal of stress release [64].

### 4.8. Combined Micromachining

Since both surface and bulk micromachining techniques possess disadvantages, a technique based on aspects of both of these micromachining techniques was developed [64]. A combination of both of these methods utilises etching techniques followed by deposition techniques which in turn create more complex structures [79].

### 4.9. Electron-Discharge Micromachining

Electro-discharge machining (EDM) is the process of machining any conductive material regardless of its chemical and mechanical properties, whilst getting thermo-electric energy as a side product [99]. Similar to EDM, micro-electro-discharge machining or electro-discharge micromachining were utilised in the fabrication of microscale features on any conductive material that gives a crucial advantage [100]. Therefore, it is frequently used in NEMS/MEMS applications [73]. It is used for the development of micro-electrodes and micro-gears [101], as well as accelerometers [102].

### 4.10. Laser Micromachining

Laser micromachining is a NEMS/MEMS fabrication technique which does not require a mask, nor any post fabrication steps [79]. Aside from not needing a mask during fabrication, laser micromachining is precise, fast and has fewer heat-affected zones [68]. In addition to its frequent use in the fabrication of biomedical technologies, laser micromachining is widely used in optoelectronics, photonics and applications utilising glass [93]. It is suitable for rapid prototyping and is commonly used in fabrication of microfluidic devices [79]. However, there is still on-going investigation into its parameter optimization for better performance [103].

### 4.11. Electrospinning

The production of nanofibers from polymer with the aid of a high electrostatic force is called electrospinning. During this process, the jet moves towards the receptor and when it is within a distance of a few nanometres with the help of Casimir force, the jet and the receptor attract which in turn attaches nanofibers to the surface of the receptor. These nanofibers are in nano-range diameters and can be used in many NEMS/MEMS-based devices [104,105].

The advantages and disadvantages of the discussed fabrication strategies are summarised in Table 1.

## 5. Clinical Applications of Nanomaterials-Based bioNEMS/MEMS

Emerging as one of the most advanced miniaturised electronics, bioNEMS/MEMS offer the indispensable potential for various analytical biology characterisation in biomedical fields. In the current research, increasing interests are directed at nanomaterials (e.g., nanocrystal, organic/inorganic nanoparticles, carbon nanoparticles and composite-nanoparticles) based nano/microsystems to sense or actuate biological components targeting diagnostics (e.g., detection early sign of diseases—cancers, cardiovascular diseases, oral diseases), monitoring (e.g., disease monitoring—diabetes, glucose and blood coagulation monitoring) therapeutics (e.g., drug delivery actuators) and cell manipulation applications [3,111,112,113]. This section highlights the clinical applications of nanomaterials-based bioNEMS/MEMS classified into diagnostic, monitoring, therapeutic and cell manipulation applications in great detail.

### 5.1. Diagnostic Applications

The ability to manufacture bioNEMS and MEMS in nano and sub-micron scales has facilitated the development of user-friendly diagnostic devices. Advancement of NEMS/MEMS in various industries such as lab-on-a-chip (LoC), miniaturised biosensors and point-of-care testing enables rapid diagnosis and monitoring of chronic diseases such as asthma, diabetes, Alzheimer’s disease, cardiovascular diseases, and cancers. Most notably, early detection of cancer biomarkers is feasible with the help of these biosensors. Table 2 summarises recent research on diagnostic and monitoring applications using bioNEMS and MEMS technology. The transition of NEMS and MEMS technology to carbon-based NEMS/MEMS (C-NEMS/C-MEMS) for cancer biomarkers detection is an attempt to increase the sensitivity of the sensing devices. Carbon-NEMS/C-MEMS involves the patterning of carbon-based materials followed by pyrolysis in an oxygen-free environment. According to Forouzanfar, et al. [114], C-NEMS/MEMS approaches offer facile fabrication of electrochemical-based sensors equipped with tailored glassy-like carbon structures (e.g., three-dimensional micropillar, microchannel, microarray) and active surfaces for functionalization due to the high presence of carboxyl groups (-COOH). This has been observed in several studies that developed label-free C-MEMS aptasensors through the introduction of carbon-rich materials (e.g., reduced graphene oxide, SU-8 photoresist) for early detection of cancer biomarkers. In both studies, electrochemical characterisation revealed a comparable linear range of detection [114,115].

**Figure 5:**
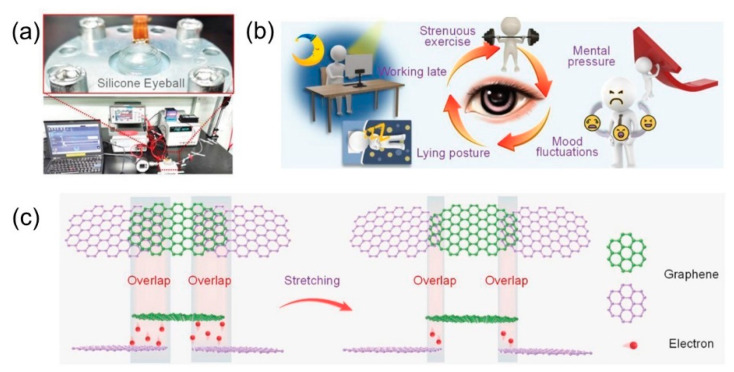
(**a**) Photomicrograph of the test platform configuration using silicon cornea, (**b**) schematic depiction of activities that could induce acute intraocular hypertension and (**c**) schematic illustration of the sensing mechanism of contact lens integrated with SAG film. Reprinted with permission from ref [117]. Copyright 2022 John Wiley and Sons.

The pressure sensor is another class of sophisticated biosensors with the ability to transduce external pressure into electrical signals. Using NEMS and MEMS technology, diagnosis and prevention of progressive vision impairment disorders such as glaucoma can be achieved by employing pressure detection. Glaucoma is known to be caused by elevated intraocular pressure (IOP) and without proper treatment, can lead to permanent blindness due to chronic optic nerve damage [116]. In most cases, glaucoma is non-detectable until a later chronic stage. A research group led by Liu, et al. [117] developed a contact lens sensor using self-assembly graphene (SAG) film and MEMS technology. The advanced sensor expressed ultra-high sensitivity towards simulated IOP fluctuation as shown in Figure 5. Furthermore, the sensor showed reliable IOP detection in vitro tests on silicone (Figure 5a) and porcine eyes. The contact lens biosensor showed enhanced sensitivity owing to the excellent strain sensitivity and desirable graphene properties with flexible overlapping morphology as illustrated in Figure 5c. To date, limited studies were invested in pressure-based biosensors using NEMS and MEMS technology despite promising findings in recent research that show the applicability of this technology for early diagnosis of glaucoma. Despite promising findings in recent research that show the applicability of this technology for early diagnosis of glaucoma, limited studies have been invested in pressure-based biosensors using NEMS and MEMS technology to date. With the estimated projection of glaucoma to reach 112 million cases by 2040, immediate interventions and research investment are required [116]. Examples of recent bioNEMS/MEMS with nanomaterials in diagnostic, monitoring and therapeutic applications are summarised in Table 2.

**Table 2:** Example studies on nanomaterials-based bioNEMS/MEMS for diagnostic, monitoring and therapeutic applications (selected studies between 2017–2022).

Technology	Type of Nanomaterials	Biomedical Application(s)	Tested Biological Samples	Outcome(s)	Application(s)	Ref
NEMS	1D photonic crystal	Cancers	-	○ Improved optical sensitivity (13 452 nm/RIU) figure of merit (FOM) (7 912 RIU^−1^) compared to existing biosensors	Label-free diagnostic biosensor (diagnostic)	[112]
	Mesoporous silica nanoparticles	Cancers	-	○ Fast delivery of drug loaded nanocarriers within 30 min without the need for external stimulation ○ Controlled release of payloads	Targeted drug delivery for cancer therapy (therapeutic)	[3]
MEMS	Titanium dioxide/tungsten trioxide (TiO_2_/WO_3_) nanocomposites	Cardiovascular diseases	-	○ Highly selective towards isoprene (breath biomarker for cholesterol abnormalities) ranging between 20 ppb (abnormal cholesterol) to 80 ppb (normal cholesterol)	Cholesterol breath analyser (diagnostic)	[118]
	Monolayer graphene	Coronavirus disease (e.g., COVID-19)	Nasopharyngeal liquids	○ Highly sensitive and label-free detection of unamplified SARS-COV-2 as low as 0.02 copies/μL in nasopharyngeal swap samples	Point-of-care testing (diagnostic)	[4]
	Biofunctionalized reduced graphene oxide	Diabetes	Human blood serum	○ Ultra-high selectivity towards glucose compared to uric acid, cholesterol and ascorbic acid ○ 4.3 times higher glucose sensitivity for 3D carbon electrodes compare to 2D carbon electrodes	Electrochemical glucose biosensor (monitoring)	[113]
	Reduced graphene oxide, gold and platinum alloy nanoparticles	Diabetes	Human sweat	○ High sensitivity with favourable glucose detection limits ○ Comparable efficiency to the commercial sensor with glucose recovery between 93 to 96%	Wearable glucose sensor (monitoring)	[119]
	Cerium oxide-polyethylene glycol-glucose oxidase nanoparticles	Diabetes	Artificial tear	○ In vivo: Detectable changes in reflection spectrum corresponded to glucose concentration in diabetic mouse group compared to non-diabetic mouse group	Contact lens biosensor (monitoring)	[120]
	Magnetic nanoparticles	Cardiovascular diseases	-	- Negligible increase in simulated body temperature (~2 °C), thus reducing risk of induced hyperthermia - In vivo catheter implantation showed good biocompatibility	Implantable catheter (therapeutic)	[118]
C-MEMS	Gold (Au) nanoparticles	Cholesterol-associated diseases	-	○ Au nanoparticles deposition facilitated selective immobilisation of cholesterol oxidase ○ High sensitivity and reliability to detect the low concentration of cholesterol	Cholesterol rapid detection (diagnostic)	[21]
	Interdigitated array nanoelectrode	Cardiovascular disease (myocardial infarction)	Human serum	Highly sensitive for cardiac myoglobin detection as low as 0.43 pg/mL	Immunosensor for cardiac Biomarker (diagnostic)	[121]
	Gold particles with dendritic nanostructures (AuNs)	Diabetes	-	○ AuNs/nanoporous sponge-like networks electrodes showed 3.8-fold higher sensitivity and 27.7-fold increase in detection limit compared to AuNs/bare carbon electrodes	Non-enzymatic glucose sensor (monitoring)	[122]

### 5.2. Monitoring Applications

Apart from diagnostic applications, these nano/microsystems have shown promising potential in various disease monitoring applications. Undoubtedly, research on monitoring glucose levels for diabetic patients receives much attention from the mainstream scientific community as diabetes is one of the top four leading non-communicable diseases behind cardiovascular diseases, cancers, and respiratory diseases. It is estimated that 693 million patients will be diagnosed with diabetes and at least half of the world’s population will remain undiagnosed by 2045 [123]. As a result, numerous studies are being directed towards early diagnosis of diabetes, monitoring of blood glucose levels and delivery of therapeutic agents (e.g., insulin). In recent years, the use of biological fluids such as whole blood, sweat and tears has shown promising outcomes for monitoring glucose levels [113,119,120] (Table 2). Monitoring glucose with enzymatic-based sensors (e.g., electrochemical and optical sensors) that rely on the detection of the glucose oxidase enzyme can be difficult. This is because of the fact that the glucose oxidase enzyme is extremely sensitive to changes in pH, body temperature, and ionic strength [124]. Despite challenges in regulating sweat behaviour in situ, current proof-of-concept studies have demonstrated the ability of their biosensors to detect and monitor glucose levels from biological sweat [119,125]. In this case, future research could look into how different real sweat conditions affect enzyme-based biosensor sensitivity and reliability.

Surgical technology has recently followed the trend of incorporating nano/micro-systems to monitor surgical performance and post-operative treatments. Because of advancements in sensor technology, real-time monitoring of various post-operative care and recovery such as fracture healing, early signs of infection determination that could potentially lead to sepsis if not properly assessed and treated, and wound recovery monitoring is now possible [126]. Implantable biosensors are also useful for many invasive implant surgeries (e.g., orthopaedic implants—total knee and hip replacements, spine implants) because they provide real-time implant monitoring or post-implantation data for improved clinical outcomes [5,126]. Anderson, Wilson and Holdsworth [5] demonstrated technical modifications to a MEMS pressure sensor that allowed them to measure changes in deformation up to 350 μm.

### 5.3. Therapeutic Applications

Recent MEMS technology advancements enable the delivery of multiple therapeutics at a predetermined time within a miniaturised implantable “pharmacy-on-a-chip” design. In 2012, Farra, et al. [127] pioneered the first human clinical trial by subcutaneously implanting a “pharmacy-on-a-chip” device to deliver an anti-osteoporosis drug an alternative anabolic osteoporosis treatment. The trial confirmed systematic delivery of the anti-osteoporosis drug (up to 20 doses per device) equivalent to the conventional subcutaneous injections. Furthermore, eight osteoporotic postmenopausal women who volunteered for the trial experienced no cytotoxicity side effects from the implanted devices and experienced minimal pain before and after implantation. Their promising findings served as a stepping stone for further development of future devices that can deliver precise dosage by opening up to 100 different reservoirs within microseconds [128]. A recent study by Li, et al. [129] developed hybridised gefitinib-loaded nanocarriers for lung tumour-targeted delivery. The hybridised nanocarrier systems embedded within the NEMS platform demonstrated a pH-responsive with prolonged in vitro drug release. Their study showed that the NEMS system was reliable for improving the delivery of gefitinib to the targeted lung tumour cells. Overall, the system has a lot of potential as a multifunctional and high-performance biomaterial for targeted lung tumour drug delivery with enhanced antitumor effect and sustained release behaviour. The latest research has demonstrated effective transmembrane delivery of drug nanoparticles through the adaptation of NEMS to the resonator technology. Specifically, Lu, Palanikumar, Choi, Huskens, Ryu, Wang, Pang and Duan [3] established an acoustic method for direct delivery of doxorubicin (DOX) across biological barriers of the cell membrane and endosomes. In this research, the delivery of DOX-loaded mesoporous silica nanoparticles to the targeted cytoplasm was navigated via controlled hypersound from the NEMS resonator. The hypersound-influenced drug delivery facilitated cellular uptake of drug nanocarriers with a size ranging between 100 to 200 nm showing the versatility to deliver a wide range of therapeutic drug nanoparticles for cancer therapy. Another interesting and impactful research niche to improve the lives of Alzheimer’s patients correlates to the in vitro screening and monitoring of Alzheimer’s therapeutic drugs containing acetylcholinesterase (AChE) inhibitors. In this instance, Chae, et al. [130] developed a low-cost graphene-based enzyme biosensor for screening and monitoring enzymatic reactions between AChE and AChE inhibitor-therapeutic drugs. As compared to conventional in vivo screening using transgenic animals or heavy dependence on high-tech research instruments to analyse physiological fluids, the low-cost graphene-based enzyme biosensor successfully screened two different AChE inhibitor-therapeutic drugs (i.e., donepezil and rivastigmine). They also observed a significant enzymatic reaction between AChE and AChE inhibitors with the ability of the biosensor to detect low up to micromolar concentrations of AChE.

Nanochannels have been employed in recent studies using NEMS technology for drug delivery systems (DDS). DDS can be divided into passive and active targeting strategies. Nanochannels are part of the passive targeting strategy by having a supersaturated drug reservoir for the passive release of drugs at the targeted sites through concentration-driven transport [131]. By carefully adjusting the size and surface chemistry of microchannels, it is possible to achieve sustained drug release. In vitro and in vivo assessments using rodents, dogs, pigs and non-human primates have validated the applicability of nanochannel NEMS technology as drug nanocarriers [132]. Since the discovery of nanochannels, several promising nanochannel innovations have been commercialised and available in the market with controlled drug release properties. Recently, NanoMedical Systems launched the nStrada platform, a nanochannel chip drug delivery technology platform. At present, the company is leading the innovation of nanochannel chips by having a refillable system with high dosing properties compared to their competitors [133].

### 5.4. Cell Manipulation

**Figure 6:**
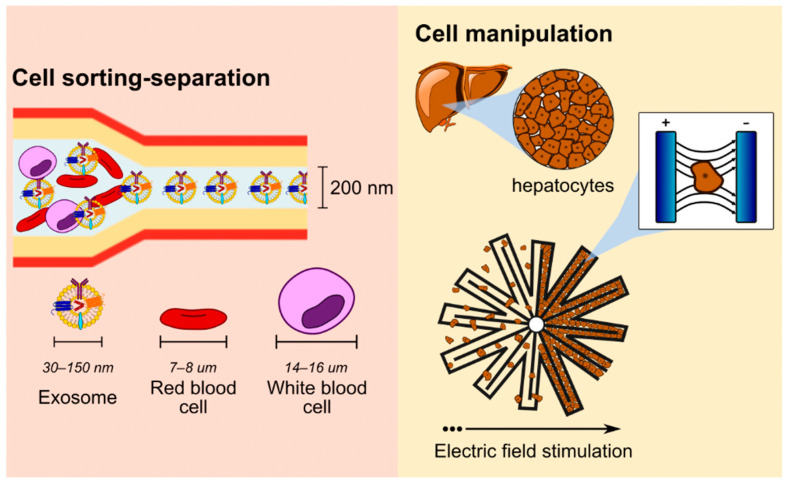
Schematic depiction of cell-sorting separation and cell manipulation adapting the NEMS and MEMS technology. Illustrations are adapted from mechanisms described by ref [6] and [111], and are not-to-scale.

The future of bioNEMS/MEMS is still blooming with limitless design possibilities considering its multifunctionalities in a myriad of biomedical applications. The technology has become an ideal tool for cell manipulation in the field of cell patterning and cell-sorting applications as illustrated in Figure 6 [111,134]. Sorting and patterning of cells or biological molecules using microchannels or nanochannels can be very useful for various clinical diagnoses, stem cell research, cell-based drug screening, and tissue engineering [135]. The guidance of cellular movement within the micro/nanochannels can be achieved via passive (e.g., channel/surface geometry, hydrodynamic forces) or active mechanisms. Active mechanisms rely on external stimulation such as dielectrophoresis (DEP), optical, magnetophoresis and acoustophoresis [111,135,136]. Integrating nano/microfluidic and LoC with bioNEMS and MEMS technology allows the fabrication of low-cost, high-throughput devices for cell sorting and separation of biological samples. The current state-of-the-art technology demonstrated promising sorting and separation of rare blood cell types (e.g., sickle-cell red blood cells), circulating cancer, or parasites from biological blood samples [136,137]. The design of microfluidic systems using nanomaterials-based bioMEMS technology can be traced back to a conceptual microfluidic bioparticle sorter conducted by Nieuwenhuis and co-workers in 2005 [138]. In this study, gold (300 nm) and chromium made up the different configurations of sorter electrodes (i.e., triangular and tetrahedral) for potential cell alignment and DEP separation. The shift towards sorting and manipulation of other biological components shows the potential of this technology for other biomedical applications. For example, Fujiwara, Morikawa, Endo, Hisamoto and Sueyoshi [6] developed a micro-nanofluidic system with nanochannels for size-sorting of exosomes, a subtype of extracellular vesicles. Classification of exosomes depending on sizes may yield more information on the exosomes’ biogenesis and a better understanding of extracellular vesicle mechanisms as potential biomarkers for liquid biopsy of cancers. In this instance, the micro-nanofluidic design developed by Fujiwara and colleagues demonstrated the ability to control the size-sorting of exosomes by manipulating the electric double layers of the integrated nanochannels.

## 6. Integrating NEMS/MEMS with IoT Applications with the Help of Nanomaterials for Biomedical Applications

Nanomaterials not only play an important role in sensing technology but also contribute to the boosting of wireless body networks and the emergence of the sixth generation (6G) mobile networks. The progress of nanotechnology to some extent, has had an impact on the size of wireless devices and led to the rise of nanodevices [139]. The integration of information from nanodevices with communication technologies, the Internet of Things (IoT) as well as the Internet of Bodies (IoB) has an enormous impact on our ability to improve human health and longevity [140]. Highly functional nanodevices for real-time monitoring and response against threats before they yield catastrophic consequences are highly dependent on modern telemedicine. However, processing power and memory will be the limitations of nanodevices due to their tiny form factors [139]. To overcome these limitations, nanodevices are incorporated into either whole or parts of nanocircuits namely nanosensor, nanoprocessor, nanomemory, nanoantenna, and nanobattery for robust technologies [140]. Adopting nanomaterials within nanocircuits not only ensures the functionality, energy efficiency, and accuracy of nanodevices while reducing their size, but also ameliorates the device properties and function itself compared to normal device size.

This section will review the role of nanomaterial in the development of nanodevice and IoT for healthcare applications.

### 6.1. Nanosensor

Nanosensors are one of the important parts of a nanodevice that are capable of sensing physical, chemical, and biological monitoring [141]. It comes in a nanoscale size, nanosensor is a miniaturised traditional bioanalytical method, such as immunoassay, chromatography, and spectroscopy, by integration of microfluidics, and microelectronics in lab-on-a-chip [142]. Nanomaterials and nanotechnology are widely used during the fabrication process of nanosensors to enhance accuracy, precision, and sensing detection [143,144]. The integration of nanosensor with IoT and body area network (BAN) technology in wearable and implantable sensors offer many advantages in providing real-time point-of-care diagnostics and therapeutics. Furthermore, the IoT and BAN platforms are considerate of patients’ health and information privacy, as well as security concerns [145]. A comprehensive table highlighting the nanomaterials based nanosensors in wearable devices and implants for various biomedical applications is provided in Table 3.

### 6.2. Nanoantenna

Antennas are an important part of wireless communication data transfer, including body area networks (BAN) and IoT. In BAN, signals from nanosensors or biosensors are transmitted to the IoT gateway or bridge by using Bluetooth (IEEE 802.15.1) and Zigbee/Xbee (IEEE 802.15.4) connectivity modules within the permitted frequency bands for medical devices according to the Industrial, Scientific, and Medical (ISM) radio band, 2.45 GHz. The IoT gateway or bridge then transmits the received signals from the nanosensors or biosensors to the cloud storage via an internet connection (Wi-fi, 4G/5G, ethernet, etc.).

The nanoantenna is a nanoscale version of radio-frequency (RF) or microwave antennas used for signal transmission and reception [152]. Desirable properties such as radiation, bandwidth, and transmission efficiency of the nanoantenna are not only maintained but enhanced with the use of nanomaterials (e.g., quantum dots, metallic nanostructures/nanoparticles, graphene, indium-doped tin oxide (ITO)-based CNT and silver-nanowires) compared to the conventional metal antenna [153,154,155]. Recently, 2D titanium carbide (MXene) coating is explored in wireless communication and offers an opportunity to produce transparent antennas [156]. To date, the nanomaterials-based nanoantenna not only improves the transmission performance of existing wireless modules and communication technologies such as Bluetooth, Zigbee/Xbee RF modules, and 5G, but also contributes to the 6G communication technology. The evolution of the 6G network has become an important point in the healthcare sector toward smart healthcare treatments that eliminate time and space barriers through patient remote monitoring and telesurgery, for instance [157].

Aside from high-speed communication, nanoantenna radiation and electromagnetic wave properties have the potential to be used for biosensing and imaging [154,158], energy harvesting [159], optical circuits [160], and in vitro and in vivo applications [161]. Table 4 summarises the benefits of nanomaterials-based nanoantennas for wireless sensing technology and healthcare applications.

### 6.3. Nanoprocessor

The processor is the heart and brain of every electronic device. It is primarily made from silicon and consists of transistors. The nanoprocessor and its core (nanocore) are constructed from intrinsically nanometre-scale building blocks and designed to be implemented with minimal logic resources to control memories for small tasks [165,166]. In IoT applications, the main feature of a nanoprocessor is the transfer of the data flows from far-end devices through the communication channels in real time. Small energy consumption and low cost yet high speed are among the important features for nanoprocessors to function as IoT nanodevices [165].

The development of the nanoprocessor is yet to surface and is still in its state-of-the-art stage [167]. However, the use of nanomaterials in processor and core fabrication demonstrates their performance ability compared to silicon-semiconductor-based materials [168]. Table 5 summarises the role of nanomaterials in reducing energy consumption, boosting processor speed and moving towards flexible read-access memory for IoT and wearable smart data storage.

### 6.4. Nanobattery

Nanobattery is not only defined based on its nanosize but uses nanotechnology in a macro-sized battery to enhance its performance and lifetime [172]. Nanobattery performance metrics include higher power density, faster charging times, and longer shelf life. Nanomaterials with different morphologies such as confined nanoparticles, nanosphere, nanopyramid, nanowires, nanostraws, nanotubes, and nanosheets with different compositions can function as an anode, cathode, separator, and electrolytes in nanobatteries [173,174]. The nanomaterials play important roles in rechargeable lithium (Li) batteries in enhancing Li storage hosting of active materials, polysulfide (PS) adsorption and conversion, oxygen reduction reaction (ORR), and oxygen evolution reaction (OER) [173].

The emergence of wearable devices has led to the development of flexible and stretchable batteries due to reasonable energy densities, being lightweight, portable, wearable, and implantable [174,175]. Carbon nanomaterials such as graphene, CNTs, and their composites have demonstrated potential for modifying the composition and fabrication of flexible electrodes [174]. Nanomaterial-based electrodes in Li-ion and lithium-sulphur batteries such as graphene/MXene heterostructures exhibit outstanding performance in improving the electrochemical performance of fast charging batteries [176,177]. The incorporation of safe and rechargeable zinc/manganese dioxide chemistry into biocompatible poly(styrene-isobutylene-styrene) (SIBS) polymer and carbon nanofiber has resulted in the development of washable rechargeable batteries. Nanomaterial-based flexible, stretchable, and washable batteries have attracted significant interest for powering systems in wearable devices, electronic skin, strain sensors, and implantable medical devices [178].

## 7. Safety and Toxicity, the Biocompatibility of Nanomaterials-Based bioNEMS/MEMS

### 7.1. Safety

Numerous regulatory bodies that evaluated the effects of nanomaterials on occupational safety and health have published their official guidelines [179,180,181,182,183,184]. Potential dangers may include the following: (i) inhaled nanomaterials may deposit in the respiratory tract and harm lung cells and tissues, (ii) certain nanomaterials have the potential to breach cell membranes and harm cellular structures and functions, (iii) risk of explosions and fires may be present in some nanomaterials because they may be pyrophoric or easily combustible.

Inhalation, dermal contact, unintentional injection and ingestion can all result in exposures, and the risk grows as exposure time and nanoparticle concentrations in the sample or air rise. The greatest exposure hazard is inhalation. Sonication, shaking, stirring, pouring, or spraying can also cause inhalation exposure, while nanomaterials suspended in a solution or slurry present a lower risk. The least dangerous nanoparticles are those that are fixed inside the matrix. Risk assessment, engineering and administrative control are recommended work practices [179]. To identify the necessary control measures and ascertain whether the implemented controls are successful in reducing exposure, a thorough risk assessment should be carried out. It is advised to use high-efficiency particulate absorbing (HEPA) filter-equipped ventilation enclosures or local exhaust ventilation that operate at negative pressure. Administrative controls consist of developing protocols for decontaminating surfaces and cleaning [184]. 

The term “nano” should be included in the descriptor, for example, “nano materials”, and should be stored in labelled containers that indicate their chemical content and form.

Building validated models that can forecast the emission, passage, modification, deposition, and uptake of nanomaterials in the environment is necessary for scientists and researchers to evaluate the safety of nanomaterials [185,186].

Because of their small size, nanoparticles found in bioNEMS/MEMS structures may be able to interact with different biological barriers in the body. This interaction could cause a toxic effect. On the other hand, drug delivery could benefit from this. However, it is crucial to comprehend whether the nanoscale carriers have any negative effects. The fate of nanomaterial during diagnosis or treatment, as well as how the particles are excreted, biodegraded, or accumulated, is crucial because accumulation can have negative long-term effects, and therefore the safety profile of nanomaterials in humans is still a significant concern [187,188].

Two categories can be used to describe safety and health hazards associated with the use of nanomaterials in bioNEMS/MEMS: (i) potential medical applications for treating diseases using nanotechnological innovations; and (ii) additional health risks associated with nanomaterial exposure [189,190].

**Figure 7:**
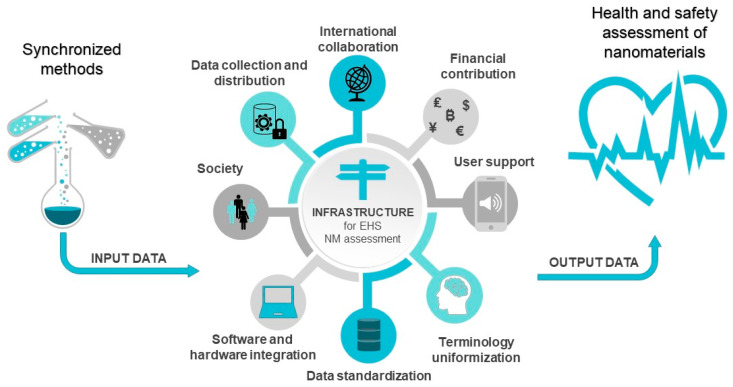
Platform for safety assessment of nanomaterials with focus on human health and the environment. EHS, environmental, health and safety; NM, nanomaterials.

It has been speculated recently that it is time for a shift in perspective when it comes to the safety evaluation of nanomaterials in bioengineering (Figure 7). The suggestion presented recently is that we should stop worrying about insurmountable problems and instead pay attention to the significant progress that has been made in past few years as new and advanced approaches have been integrated into nanosafety research [191]. To use the newly developed techniques for the risk and hazard evaluation of biological nanomaterials, we need a paradigm shift in the way that nanosafety assessment is carried out.

### 7.2. Toxicity

There are many worries about the potential toxicity of nanoparticles. Suitable regulations for their use and evaluation are regularly amended and the recently adopted ISO standard 10993-22 is devoted to the biocompatibility assessment of nanomaterials used in medical devices [192]. Cell survival has been found to be influenced by nanoparticle features such as size, surface charge, dispersion state, matrix composition, surface functionalization, and protein corona formation [193]. These parameters must be mastered in order to conduct a biocompatibility assessment using the appropriate methodology. Numerous assays are used to determine the toxicity of the nanostructures because there are more nanomaterials and nanoparticles every day [185]. These tests are categorised into two main groups: in vitro and in vivo tests. Table 6 summarises the cytotoxicity methods recently used in nanomaterials toxicity assessment along with the main effects observed during evaluation in specific cell lines and tissues.

#### 7.2.1. In Vitro Assessment of Nanomaterials Toxicity

##### Cytotoxicity Assays

In vitro cytotoxicity assays are among the gold standard technique to assess the cytotoxicity of nanomaterials.

1. Microculture tetrazolium assay

The colorimetric microculture tetrazolium assay is intended and widely used for a qualitative evaluation of the nanomaterial cytotoxicity [208]. It is a non-radioactive assessment technique that measures cell viability using the metabolic activity of the mitochondria. When it comes to cytotoxicity testing of nanomaterials-based NEMS/MEMS MTT test has been employed in various systems. Nano-crystalline Zr x -Cu100x thin films with thicknesses ranging from 50 to 185 nm were assessed in terms of their cytotoxicity. As determined by the MTT assay, Zr-Cu thin film is suitable as a bio-coating for minimally invasive medical devices because it did not cause cytotoxicity in osteoblast cells [194]. A graphene field-effect transistor-based artificial synapse was used for the MTT test of the bioMEMS system, and the results showed that the cell viability (against HK-2 cells) shows no decrease with the increased concentration, which suggests that the silver gel is a biocompatible material [195].

The MTT test has been employed in the initial biocompatibility testing of a peristaltic micropump that is wirelessly controlled in murine inner ear drug delivery, where a silicon MEMS-based 32-kHz oscillator was used [209]. A biocompatibility study was conducted, and the outcomes showed that the micropump parts passed important biocompatibility tests after a month a micropump prototype was implanted without any signs of infection or inflammation. According to these findings, these particular micropump parts passed important biocompatibility tests, indicating that they were appropriate for translational applications such as subcutaneous implantation in both humans and animals.

2. Trypan blue exclusion assay

Trypan blue testing has recently been employed to examine the cytotoxicity of various coatings used with resin 3D printing. It has been demonstrated how multiple procedures can be coupled to offer better biocompatibility, which in turn increases accessibility to 3D-printed BioMEMS manufacturing. Any combination of the post-process treatments resulted in viable cultures (above the 85.00% threshold) for HL-1 cells. Similar performances of above the 85% threshold were observed for all of the coatings (PDMS, PS, SU-8, Au, and Medco/PET), with PDMS outperforming the others [197]. Cytotoxicity in encapsulated particles in aqueous ferrofluid droplets was examined, and a magnetic field was used to separate the encapsulated particles from the empty droplets. Trypan blue test results showed that 86% of cells were still alive after 2 h when compared to the control, showing that the ferrofluid used in the experiments had little impact on the viability of the cells. These findings suggest that the proposed method for sorting empty droplets in biochemical assays is appropriate [210]. Trypan blue testing demonstrates also that both the silicone-modified polyurethane films and nanofibers caused less toxicity in contact with cells. In contrast to the positive control, the cells can maintain their spreading shape and discrete intracytoplasmic granules without experiencing cell lysis, which can be considered as survival and the morphological grade of cytotoxicity is supposed to be zero [196].

3. Clonogenic assay

The carbon nanoparticles’ relative toxicities are described in a recent review [198]. It has been shown that the small size of nanoparticles permits them to penetrate through harmful biological barriers. Depending on the type of analysis and models, negative consequences are highlighted.

4. Apoptosis assay

Apoptosis, or programmed cell death, has found significant application potential in the evaluation of nanotoxicity. Analysis of the DNA damage caused by polysaccharide-surface functionalised (coated) and non-functionalised (uncoated) silver nanoparticles in mammalian cells, including mouse embryonic stem cells and mouse embryonic fibroblasts, revealed that severe DNA damage could cause the cells to undergo apoptosis. The enhanced activity of the apoptosis markers caspase-3 and caspase-9 provide further proof that silver nanoparticle therapy activated the apoptosis pathway in Drosophila melanogaster larval tissues [199].

5. DNA laddering

Various genotoxic effects of graphene nanoparticles were hypothesised based on the recent literature findings [200,201,202]. There are not many investigations on graphene genotoxicity caused by direct interaction with DNA as of now [200,202,203]. It has been well documented that DNA damage results from oxidative stress brought on by graphene nanoparticles [200,203]. Studies on other forms of indirect genotoxicity, including epigenetic toxicity, inflammation, as well as autophagy, mostly concentrate on the genotoxic effects brought on by graphene. There is not much data on the mechanisms underlying this impact [211]. The inherent physicochemical characteristics of graphene nanoparticles (such as surface functionalization or coatings), exposure dose or durations, and their destination in organisms or the surroundings will all affect how genotoxic they are [200,212].

6. Caspase assay

Caspase assay has recently been used for determining the molecular basis of hepatic toxicity brought on by mesoporous silica nanoparticles (MSNPs) both in vitro and in vivo. The findings showed that these nanoparticles caused liver inflammation, hepatic cell pyroptosis, and hepatotoxicity. These findings shed new light on the hepatotoxicity caused by MSNPs and show that the NLRP3 inflammasome, pyroptosis, and reactive oxygen species (ROS) are effective targets for raising biocompatibility and lowering the potential toxicity of MSNPs. To avoid the adverse effects of MSNPs in future biomedical applications, more study is required to assess the association between hepatotoxicity and the sizes or surface chemical modifications of MSNPs in vivo [204].

7. Comet assay

The possibility for DNA damage by nanomaterials is the most significant of their possible impacts. The development of standard techniques for genotoxicity detection is a key objective of the EU project NANoREG. One of the proposed techniques mentions the use of comet assay as a tool to find DNA strand breaks that have been induced. Using two different human lung epithelial cell lines, eight different nanoparticles—titanium dioxide (TiO_2_) NPs, silica dioxide (SiO_2_) NPs, zinc oxide (ZnO) NPs, cerium dioxide (CeO_2_) NPs, silver NPs, and multi-walled carbon nanotubes were examined [205]. The outcomes supported the usefulness of the comet assay to identify nanomaterial’s potential for genotoxicity. The findings suggest that the majority of the nanomaterials had mild to considerable genotoxic effects, demonstrating the applicability of the cell line to assess a material’s genotoxic potential.

8. TUNEL assay

Rat liver and intestinal NLRP3 inflammasome, oxidative stress, and apoptosis were studied in vivo and in vitro using TiO_2_ NPs [206]. Titanium dioxide nanoparticles increased cytotoxicity, oxidative stress, and apoptosis rate in a dose-dependent manner. Additionally, TiO_2_ NPs increased the gene expression of the inflammation and apoptotic pathways in the liver and gut. The liver and gut experienced morphological alterations as a result of TiO_2_ NPs. TiO_2_ NPs may cause oxidative stress, inflammation, and apoptosis, which may have deleterious effects on the structure and operation of the liver and the intestines.

9. Annexin V and Propidium iodide (PI)

From the standpoint of cytotoxicity, graphene oxide–silver nanoparticles (GO-AgNPs) have attracted a lot of interest for their potential in biomedical applications. However, it is unknown if GO-AgNPs are hazardous to people and animals. In caprine foetal fibroblast cells, GO-AgNP-mediated cytotoxicity and epigenetic modification status were found. It was completed using the annexin V/PI assay [207]. The results showed that GO-AgNPs cytotoxicity is dose-dependent. By decreasing cell viability, producing ROS, boosting lactate dehydrogenase and malondialdehyde leakage, and enhancing the expression of pro-apoptotic genes, GO-AgNPs significantly increased cytotoxicity. DNA hypomethylation and DNMT3A expression were both induced by GO-AgNPs. The prospective applications of GO-AgNPs in biomedicine should be reassessed because they increase the production of ROS, induce apoptosis, and result in DNA hypomethylation.

##### Oxidative Stress Assays

1. 2,7-Dichlorodihydrofluorescein (DCFH) assay

When DCFH is used in an assay, ROS transforms it into the fluorescent substance 2,7-dichlorofluorescein, which is then identified by fluorimetry. Since DCFH is readily oxidised by several ROS functional groups, it is highly favoured as a cytotoxicity assay. This fluorogenic dye assesses the activity of ROS, including hydroxyl, peroxyl, and others. When a dye enters cells, esterases deacetylate it into a non-fluorescent substance. The latter is transformed into dichloro-dihydro-fluorescein by reactive oxygen species, which can be detected with a fluorometer, a flow cytometer, or a fluorescence microscope with an excitation spectrum at 485 nm and an emission spectrum at 535 nm [213]. For quantitative determination of H_2_O_2_ and other hydroperoxide group members such as tert-butyl hydroperoxide, DCFH is a regularly used assay. For nanoscale zinc oxide particles, used in the human lung adenocarcinoma cell line DCFH assay demonstrated that these NP cause apoptosis by raising intracellular ROS levels [199].

2. Lipid peroxidation assay

Lipid peroxidation has been connected in some cases to the cytotoxicity of carbon-based nanomaterials (CBNMs) [198]. The small size of CBNMs permits them to penetrate harmful biological barriers. One of the most significant toxicity mechanisms of NPs is the generation of ROS, which causes oxidative stress, inflammation, lipid peroxidation, and damage to the proteins, cell membrane, and DNA. Oxidative stress is defined as an imbalance between the biological system’s capacity to remove reactive intermediates (superoxide radical anions and hydroxide ions) and the amount of ROS produced. This imbalance may be caused by either an increase in ROS production, a decrease in the cell nucleus defence mechanisms, or a combination of both.

#### 7.2.2. In Vivo Assessment of Nanomaterials Toxicity

A variety of techniques, including biodistribution, clearance, serum chemistry, and histopathology, animal models such as mice and rats are frequently used.

##### Biodistribution

The biodistribution and nanotoxicity of NPs are governed by their physiochemical properties. Following the administration of nanomaterials, the presence of nanomaterials in key organs or tissues such as the lung, liver, kidneys, heart, brain, pancreas, fat, and muscle is estimated. Then, physical detection techniques can be used to monitor the distribution of NPs in these organs. By using instrumental neutron activation analyses for gold composite NPs in animal tumour models, it was demonstrated that composite nanodevices might target specific organs based on their size and/or surface charge [199]. The biokinetics and biodistribution of nanomaterials delivered through the respiratory system differ from those following intravenous exposure. The biokinetics of intravenously administered nanomaterials does not serve as a substitute for exposure through the lungs or mouth [214]. Rats were used to evaluate the biokinetics and biodistribution in diverse organs over the course of 1 h to 28 days using radiolabelled TiO_2_ NPs. The liver accumulated the most titanium following intravenous injection, followed by the spleen, skeleton, and blood after 1 h. Thereafter, the blood’s volume rapidly shrank, but the distribution of titanium in the other organs and tissues remained stable until day 28 [215].

##### Clearance

By analysing the excretion and metabolism of NPs at a certain time point after NP therapy, the clearance of NPs is investigated. Their transit to the liver and kidney was discovered to be significantly constrained [199]. Nanomaterials that enter the vascular system connect with blood cells and are then disseminated to the organs on the periphery. Their size, surface structure, and changes all affect how they are distributed to peripheral organs and how they are cleared. Due to their prolonged circulation duration, nanomaterials that are not effectively eliminated from the body are more likely to interact with cells, tissue, and organs and accumulate there. Different pathways might be used by the body to remove nanoparticles. Although renal clearance is the most efficient excretion method, many nanomaterials cannot be cleared via this method due to size limits (6–8 nm) [214]. Instead, they undergo biliary excretion, where they are broken down by the liver and eliminated through the gastrointestinal tract. As nanoparticles trapped in the mucus are normally carried to the pharynx and then swallowed, mucociliary clearances in the respiratory system can be closely associated with gastrointestinal clearance. Nanomaterials that have been ingested by mononuclear macrophages can stay for a very long time, trapped within the reticuloendothelial system, while elimination via the kidneys and liver can take place over a timescale ranging from 30 min to a few days.

##### Serum Chemistry

To evaluate nanotoxicity, alterations in serum chemistry after NPs administration are analysed. Blood urea nitrogen, creatinine, glucose, total protein, albumin, globulin, albumin to globulin ratio, sorbitol dehydrogenase, alanine aminotransferase, alkaline phosphatase, and creatine kinase are the parameters that are determined during serum chemistry tests. Red blood cell count, haemoglobin, packed cell volume, mean red cell volume, mean red cell haemoglobin, mean red cell haemoglobin concentration, white blood cell count, differential white blood cell count, and platelet count are all variables that must be determined during a haematological analysis [199].

##### Histopathology

Histopathological analysis of NP-exposed organs, including the heart, lungs, spleen, liver, kidneys, and eyes, is another method for determining nanotoxicity. After exposure to nano-ferric oxide, follicular lymphoid hyperplasia with inflammatory cells gathered around bronchia was discovered. Alveolar walls thickened after NP therapy, indicating the establishment of fibrosis. The pulmonary alveoli were discovered to contain the macrophage-phagocytosed particles. The pulmonary alveolus contained inflammatory cells such as neutrophils, lymphocytes, and eosinophils [199].

### 7.3. Biocompatibility

The biological risk has to be calculated as part of risk management, by standards, for the medical device to be regarded as biocompatible [216]. The manufacturer is in charge of assessing the biocompatibility of a finished device, and they should do so as early in the product life cycle as possible to plan a strategy to meet regulatory requirements. An updated version of ISO 10993-1 that stressed the value of learning more about the device was released in August 2018 [217]. Not all biological effects should be evaluated by biological testing, according to the new version of the standard. If a valid reason can be provided, some tests can now be waived. Although there is a truly impressive number of articles describing bioNEMS/MEMS systems for various medical indications, not all of them provide biocompatibility data, much less according to the ISO 10993 standard. However, some studies accurately reported the biocompatibility tests that were actually conducted with the design of NEMS/MEMS systems.

In the literature, less optimistic views on this topic can be found regarding the rapid translation of bioNEMS/MEMS systems into clinical practice. In a recent review, Burton [218] suggests that findings demonstrate that clinical translatability is hampered by insufficient consideration of the biocompatibility of nanorobots with human in-vivo environments in upstream development processes. The design focus is undoubtedly amplified by the absence of meaningful integration with multidisciplinary downstream knowledge.

Utilising MEMS technology, the vaccine-coated solid microneedle patch was created via the process of silicon microneedle arrays using wet chemical etching, inductively coupled plasma reactive ion etching and UV lithography [219]. This combination of vaccine formulation and microneedle to fabricate the delivery device was evaluated in vitro and the material under test exhibited no cytotoxic effects and complied with ISO 10993-5 biocompatibility requirements.

In general, we can take parts of cells and partially make them function outside the organism using nanotechnology. Adenosine triphosphate, a high-energy phosphate particle used to store and deliver energy for work inside living organisms, expands as the engines move. The devices might be on a scale that is comparable to a single molecule. Such devices would malfunction as a result of wear. Particularly for man-made nanomachinery used in physiological conditions, tribological considerations, together with biocompatibility concerns in the design are critical [220].

For implants and encapsulations, biocompatibility should be assessed from two different perspectives [221]. The first is surface biocompatibility, which states that the coating must be chosen to reduce the foreign-body reaction, prevent any significant post-operative inflammation, and prevent the diffusion of harmful materials. In addition to cytotoxicity testing, non-biocompatible chemical groups can be detected by changes in cell shape and metabolism. The criteria for assessing a medical device’s biocompatibility are part of the ISO 10993 standards. The mechanical compatibility between the encapsulated implant and the surrounding tissue is the focus of the second form of biocompatibility, known as structural biocompatibility. To lessen implant insertion injury, particularly to the central nervous system, new materials and techniques are required. In vivo tests need to be performed to evaluate this property.

Another aspect of biocompatibility that plays a vital role in biomedical applications, as well as the repurposing of food waste to fabricate nanomaterials for potential applications in bioNEMS and MEMS is non-toxic and high nutritional values. As discussed previously, nanomaterials are one of the most researched materials in drug delivery. Because of their adaptability and numerous intriguing qualities, including controlled release, blood stability, non-immunogenicity and non-toxic nature, biodegradable polymer-based nanoparticles are used in innovative drug delivery methods [222,223]. Colloidal drug carriers such as liposomes, emulsions, micelles and nanoparticles have shown promising characteristics as future medications that can cross the blood-brain barrier. Similar to this, colloidal systems are employed to control the rate of medication release at targeted areas (i.e., tissues or organs). There are myriad nanomaterials (e.g., silver nanoparticles, gold nanoparticles, carbon nanotubes, laponite nanoplates, polymer-based nanoparticles) that paved the way towards greener MEMS and NEMS [224,225].

Recently, the World Health Organisation also created some guidelines with advice on ethics and good laboratory practices for how to better protect workers from the possible risks of dangerous nanomaterials. Anyone can share their view of the ethics of nanotechnology in several different forums. The opinions of these populations tend not to be based on the vocabulary of the professional ethicists. Researchers, engineers, philosophers and policymakers should communicate and provide their ideas in a similar and simple language that is clearer and more effective [226].

## 8. Challenges and Conclusions

This review summarises the most recent advancements in bioNEMS and MEMS based on nanomaterials for biomedical applications. We discussed the critical nanomaterial selections and desirable properties (for example, mechanical, optical, and electrical) as biosensors, actuator systems, drug nanocarriers, and other biomedical applications. The evolution of fabrication strategies from traditional techniques (such as bulk and surface micromachining) to more advanced techniques such as photolithography and electrospinning was thoroughly discussed. According to the most recent literature review, the incorporation of nanotechnology into NEMS and MEMS has demonstrated undeniable potential in clinical applications (i.e., diagnostic, monitoring, therapeutic applications, cell manipulation) with dependable functionality in vitro and in vivo. As a result, more groundbreaking discoveries and innovative bioNEMS/MEMS-based platforms are expected to be translated from bench to bedside in the future. Despite thriving research in the field of nanomaterials-based bioNEMS/MEMS in recent years, bioNEMS/MEMS for real-world human applications is still in its infancy. Many ideas remain unexplored, and numerous challenges in this field remain unsolved. As such, some of the major challenges are highlighted as follows. One of the major challenges associated with this technology is the scaling up of materials for commercialisation. In most cases, high-quality nanomaterials involved precise synthesis routes under harsh conditions with heavy reliance on sophisticated instrumentations [9]. Furthermore, expensive chemicals required for nanomaterial synthesis could further hinder the scaling-up process due to projected increased production costs. An attempt to reduce production costs by switching to cheaper materials may jeopardise the quality of nanomaterials, thus the overall functionality and reliability of the bioNEMS/MEMS platforms.

A paradigm shift towards upcycling food waste to create innovative nanomaterials for future bioNEMS/MEMS devices will be a crucial step for sustainable food waste management. The food waste-to-nanomaterials conversion strategy is anticipated to reduce carbon footprints and greenhouse gas emissions (e.g., methane and carbon dioxide) to the atmosphere. Food wastes are rich in valuable nutrients from organic to inorganic biomolecules (e.g., flavonoids, phenolic and alkaloids compounds) with a proven sustainable synthesis of food waste-mediated nanomaterials such as silver and gold nanoparticles. These high-value engineered nanoparticles have shown potential for various biomedical applications due to their non-toxicity, better biocompatibility, and green synthesis properties. Furthermore, the conversion of food waste materials into useful nanomaterials could potentially reduce the cost of source materials, eventually controlling the end-user price [28,44]. Regardless, pre-processing of food waste could contribute to higher overhead costs, thus requiring deliberate strategies to overcome it. Food waste valorisation and zero-waste strategies have opened up exciting research avenues in the NEMS and MEMS applications with the foreseeable future of green device technology.

Taking a glimpse into integrating nanotechnology and NEMS/MEMS technology to IoT applications, limitless opportunities and challenges lie ahead of this exceptional idea. The current state-of-art knowledge proved that there is potential for further research and scientific breakthroughs [168]. Despite the opportunities of IoT technology in improving human health and longevity through rapid disease diagnosis and real-time treatment monitoring. Upcoming research direction is anticipated to delve into proof-of-concept experiments and application research in the next decades is of paramount importance. Above all, challenges associated with nano biodevices into IoT systems can be classified as the followings [140]:

1. Connectivity

In a telehealth system, any disruption in data transmission could have devastating patient consequences. IoT device connectivity depends on the transmission coverage of its wireless modules such as RF module, Bluetooth, and Wi-Fi. For that reason, wireless and internet connectivity in the healthcare setting must be strengthened with ultra-high reliability and low-latency communication networks to prevent connectivity problems during an emergency situation.

2. Data continuity

Nanodevices such as wearable and medical implants are highly dependent on the battery’s life to ensure their functionalities, efficiency, and performance [227]. Frequently charging the devices may disturb data continuity while direct charging of wearable devices exposes patients to an electrical hazard. The incorporation of nanomaterials in maximised nanobattery runtime and the emergence of wireless energy harvesting through nanoantennas potentially provide uninterrupted access to essential patient data.

3. Compliance

Healthcare IoT is subject to evolving regulations that vary by country. For example, US Food Drug Administration (FDA), CE marking for European Union, Therapeutic Goods Administration (TGA) Australia, and Medical Device Authority (MDA) Malaysia are compliance bodies that outline the need for device makers, pharmaceutical companies, and other players to ensure the safety, efficiency, and reliability of medical devices including IoT device for healthcare applications.

4. Coexistence

Other than IoT nanodevices for healthcare applications, there are many existing IoT devices currently available that potentially compete for connectivity. The existence of many IoT nanodevices in specific and limited spaces potentially leads to signal interference between devices that causes connectivity failures or corrupted data.

5. Cybersecurity

Health Insurance Portability and Accountability Act (HIPAA) sets the standard for sensitive patient data protection. Under HIPAA, companies that deal with protected health information (PHI) must comply with the physical, network, and process security measures outlined in the HIPAA. Although data collection using IoT devices commonly contain basic information such as patient ID and physiological information, the health and information privacy of patients, and security concerns remain important. Security breaches potentially expose patient information to irresponsible parties.

Regardless, prospects of NEMS and MEMS for engineering-based applications have been positive leading to rapid translation of this technology to biomedical fields. In closing, it is anticipated that this comprehensive review article will enlighten novel research directions and inspire ideas for further exploration of nanomaterials-based bioNEMS/MEMS for biomedical applications in the near future.

## Figures and Tables

**Table 1:** Advantages and disadvantages of bioNEMS/MEMS fabrication strategies.

Technique	Advantages	Disadvantages	Ref
Photolithography	○ Strong chemical stability ○ Adequate adhesion ○ Good photosensitivity	○ Resolution limited to the wavelength of the used light	[63,65]
Electron Beam Lithography	○ No mask used ○ Precise control over energy and dose of the electron beam ○ Accurate registration over small areas on a wafer ○ Lower defect densities	○ Low exposure speed ○ Costly method ○ Resolution is limited to the diffraction of electron beam in solids	[64,73,74]
Scanning Probe Lithography	○ High resolution method ○ Electrons have lower energy in comparison to EBL ○ Low cost ○ 3D patterning ○ High quality patterning	○ Relatively slow method	[64,75,76]
Soft Lithography	○ Cost-effective ○ Does not require much expertise or sophisticated equipment	○ Slow ○ Other methods are commonly preferred	[64,106]
Nanoimprint Lithography	○ High resolution (better than 10 nm) ○ High efficiency	○ Mechanical misalignments are common ○ Defectivity and template life are difficult to control	[74]
Bulk Micromachining	○ Low cost ○ Etched surface is smooth ○ Good for making holes in MEMS	○ No layering on the same wafer	[71,79,92]
Surface Micromachining	○ Simple method ○ Low cost ○ Convenient formation of multilayer systems	○ Due to thin layers, bending is frequent ○ Post processing is needed for stress relieving	[64,107,108]
Combined Micromachining	○ Decreased fabrication cost; improves yield ○ Low cost ○ Guarantees electrical reliability	○ Post processing is needed	[79]
Electron-discharge Micromachining	○ Fabrication on any conductive material	○ Only used on conductive material	[99]
Laser Micromachining	○ No mask required ○ No post fabrication steps ○ Precise, fast ○ Few heat-affected zones ○ Good for rapid prototyping	○ Micro cracks ○ Shock wave surface damage	[68,79,109]
Electrospinning	○ Suitable for testing ○ Simple set up ○ High modulative capability ○ High productivity	○ Great nanofiber diameter distribution	[105,110]

**Table 3:** Examples of nanomaterials-based nanosensors in wearable devices and implants.

Application	Types of Nanosensor	Advantage(s)	Ref
Wearable cardiovascular monitoring	○ MEMS pressure sensor array ○ Electrochemical ○ Optical ○ Pressure ○ Paper-based	○ IoT platforms in cardiovascular monitoring potentially reduce morbidity and mortality statistics due to early diagnosis and response	[143,146]
Human steroid Hormone sensing	○ Nanoslot array-based terahertz molecule-specific sensor chip ○ Implantable sensor	○ Discrimination and quantification of trace amounts of steroid hormones in biological specimens to elucidate changing expression and prevention of endocrine disorders	[147,148]
Artificial auditory system	○ Piezoresistive ○ Hydrogel ○ Nanocomposites	○ Closely mimic the biological cochlea for hearing disorder due to ageing	[149]
Smart electronics textile for wearable body sensor	○ Carbon-based nanomaterials (CNT) ○ Reduced graphene oxide (rGO)	○ Real-time body physical, chemical and electrophysiological sensing (strain, pressure, temperature, sweat, electrocardiogram (ECG), electromyography (EMG) and etc.)	[150]
Ocular applications (contact lens)	○ Graphene contact lens electrodes	○ The graphene electrodes demonstrate high-efficacy measurements of various kinds of electroretinography without corneal irritation for ocular application	[144,151]

**Table 4:** Examples of nanomaterials-based nanoantenna and its advantages for wireless communication.

Types of Nanoantenna	Nanomaterial(s)	Advantages(s)	Ref
Flexible and low-cost patch antenna	○ MXene (Ti_3_C_2_T_x_)	○ A functional nanomaterial-based antenna that serves the dual purpose of sensing and communication ○ High potential for based wireless sensing	[162]
Circular patch nanoantenna	○ Graphene	○ Graphene nanoantenna enables high miniaturization ○ Ultra-wideband (UWB) behaviour ○ Easy reconfiguration	[163]
Graphene plasmonic Terahertz (THz) antenna	○ Graphene	○ The silicon-based substrate on graphene THz antennas demonstrates good performance in reflection coefficient, impedance bandwidth, front-to-back ratio and directivity and promising for THz short-range communication, sensing and biomedical applications	[164]
ITO-based CNT coated transparent nano E-shape patch antenna	○ ITO-based CNT	○ ITO-based CNT nanoantenna has better performance in terms of broad bandwidths, reasonable radiation efficiency, sufficient gain, lightweight and compactness ○ Suitable for satellite communication that is potentially used for telehealth in rural or areas with limited mobile communication network	[153]
Half-wave dipole MXene antenna for RF devices	○ 2D titanium carbide (MXene) coating	○ MXene coating offers a transparent antenna for various classes of RF for IoT applications, portable, flexible and wearable electronic devices	[156]
Optical dipole nanoantenna	○ Cadmium selenide quantum dots	○ Intensified Terahertz radiation for time-domain spectroscopy for medical imaging applications	[154]

**Table 5:** Nanomaterials and selected applications of nanoprocessors for future biomedical applications along with their advantages.

Application(s)	Nanomaterial(s)	Advantage(s)	Ref
Energy consumption	○ Carbon nanotube field-effect transistor (CNFET)-based digital circuits	○ The CNFET that designed and fabricated using industry-standard design flows and processes overcoming nanoscale imperfections at macroscopic scales across full water substrates ○ Offer energy-efficiency benefit	[168]
Interconnect wires in the processor core	○ Integrating graphene on the interconnect wires	○ Increase about 8% of speed boost and contribute to 12% of energy saving	[169]
High-performance resistive read access memory (RRAM)	○ Graphene oxidised with a perpendicular oxidation gradient	○ Offers a high on-off current ratio of ~105, long-term retention of ~106 s, reproducibility over 104 cycles and long-term flexibility at a bending strain of 0.6% ○ Demonstrate great potential in wearable smart data-storage devices	[170]
Complementary metal-oxide-semiconductor (CMOS) oscillators	○ Graphene- silica (Si) CMOS hybrid circuit	○ Introduces graphene in Si CMOS has ameliorated voltage swing and switch ability of Si CMOS circuit in a modern electronics system	[171]

**Table 6:** Nanomaterials and cytotoxicity assay together with the main results of toxicity assessment.

Test	Nanomaterial	Toxicity Assessment	Ref
MTT	Zr x -Cu100x	No toxicity in osteoblast cells	[194]
MTT	Graphene	Biocompatible against HK-2 cells	[195]
MTT	Silicon MEMS	No signs of infection or inflammation	[196]
Trypan blue exclusion assay	PDMS, PS, SU-8	>85% cells viability	[197]
Clonogenic Assay	Carbon	>50 cell colony formation	[198]
Apoptosis Assay	Ag	DNA damage leading to apoptosis	[199]
DNA laddering	Graphene	DNA damage due to oxidation stress	[200,201,202,203]
Caspase Assay	Mesoporous Si	Liver inflammation, hepatotoxicity	[204]
Comet Assay	TiO_2_, SiO_2_, ZnO, CeO_2_, Ag, MWCNT	Mild to considerable genotoxic effect	[205]
Tunnel Assay	TiO_2_	Increased gene expression of the inflammation and apoptotic effect	[206]
Annexin V and Propidium iodide	GO-Ag	Increased production of ROS	[207]
Lipid peroxidation Assay	Carbon based	Generation of ROS, inflammation, damage to the proteins	[198,199]

## Data Availability

Not applicable.

## Abbreviations

**2D**—two-dimensional, **3D**—three-dimensional, **4G**—fourth generation, **5G**—fifth generation, **6G**—sixth generation, **AChE**—acetylcholinesterase, **Ag**—silver, **AuNPs**—gold nanoparticles, **AuNs**—gold nanostructures, **BAN**—body area network, **bioNEMS/MEMS**—biomedical nano-/micro-electro-mechanical systems, **CBNMs**—carbon-based nanomaterials, **CeO_2_**—cerium oxide, **CNT**—carbon nanotube, **CNTFET**—carbon nanotube field-effect transistor, **C-MEMS**—carbon micro-electro-mechanical systems, **CMOS**—complementary metal-oxide semiconductor, **COOH**—carboxyl groups, **Cr**—chromium, **Cu**—copper, **DCFH**—2,7-dichlorodihydrofluorescein, **DDS**—drug delivery system, **DEP**—dielectrophoresis, **DNA**—deoxyribonucleic acid, **DNMT3A**—DNA (cytosine-5)-methyltransferase, **DOX**—doxorubicin, **EBL**—electron beam lithography, **ECG**—electrocardiogram, **EDM**—electro-discharge machining, **EHS**—environmental, health and safety, **EMG**—electromyography, **FDA**—Food Drug Administration, **FET**—field-effect transistor, **GaN/AIN**—gallium nitride/aluminium nitride, **GFET**—graphene field-effect transistor, **GO**—graphene oxide, **H_2_O_2_**—hydrogen peroxide, **HEPA**—high-efficiency particulate absorbing, **HIPAA**—Health Insurance Portability and Accountability Act, **HK-2**—human kidney-2, **HL-1**—mouse cardiac cell line, **ICP-RIE**—inductively coupled plasma-reactive ion etching, **ID**—identity, **IL-6**—interleukin-6, **IoB**—Internet of Bodies, **IOP**—intraocular pressure, **IoT**—Internet of Things, **ISM**—Industrial, scientific and medical, **ITO**—indium-doped tin oxide, **ISO**—International Organisation for Standardisation, **JFETs**—junction field-effect transistors, **Li**—lithium, **LOC**—lab-on-a-chip, **LOD**—limit of detection, **MDA**—Medical Device Authority, **MEA**—microelectrode arrays, **MEMS**—micro-electro-mechanical systems, **MWCNT**—multi-welled carbon nanotube, **MOSFET**—metal-oxide semiconductor field-effect transistor, **MTT**—(3-(4,5-dimethylthiazol-2-yl)-2,5-diphenyltetrazolium bromide, **MXene**—titanium carbide, **NEMS**—nano-electro-mechanical systems, **MSNPs**—mesoporous silica nanoparticles, **NM**—nanomaterial, **OER**—oxygen evolution reaction, **ORR**—oxygen reduction reaction, **PDMS**—polydimethylsiloxane, **PEG**—poly(ethylene glycol), **PHI**—protected health information, **PLA**—polylactide, **PS** -polysulfide, **SIBS**—poly(styrene-isobutylene-styrene), **m-SPL**—mechanical scanning probe lithography, **NIL**—nanoimprint lithography, **RF**—radio-frequency, **rGO**—reduced graphene oxide, **RIU**—refractive index unit, **ROS**—reactive oxygen species, **SAG**—self-assembly graphene, **SARS-CoV-2**—severe acute respiratory syndrome coronavirus 2, **Si**—silica, **SiO_2_**—silica dioxide, **SPL**—scanning probe lithography, **SPR**—surface plasma resonance, **TGA**—Therapeutic Goods Administration, **TiO_2_**—titanium dioxide, **t-SPL**—thermal scanning probe lithography, **UV**—ultraviolet, **UWB**—ultra-wideband, **WO_3_**—tungsten trioxide, **ZnO**—zinc oxide, **Zr**—zirconium.
